# Activation of the brain during motor imagination task with auditory stimulation

**DOI:** 10.3389/fnins.2023.1130685

**Published:** 2023-03-15

**Authors:** Long Li, Yanlong Zhang, Liming Fan, Jie Zhao, Jing Guo, Chenxi Li, Jue Wang, Tian Liu

**Affiliations:** ^1^The Key Laboratory of Biomedical Information Engineering of Ministry of Education, Institute of Biomedical Engineering, School of Life Science and Technology, Xi’an Jiaotong University, Xi’an, China; ^2^Key Laboratory for Artificial Intelligence and Cognitive Neuroscience of Language, Xi’an International Studies University, Xi’an, Shaanxi, China

**Keywords:** auditory stimulation feedback, mismatch negative (MMN), inter trial phase locking consistency (ITPC), EEG, motor imagery (MI)

## Abstract

**Introduction:**

Auditory stimulation is one of the most important influence factors in the cognitive process. It is an important guiding role in cognitive motor process. However, previous studies on auditory stimuli mainly focused on the cognitive effects of auditory stimuli on the cortex, while the role of auditory stimuli in motor imagery tasks is still unclear.

**Methods:**

In order to explore the role of auditory stimuli in motor imagery tasks, we studied the EEG power spectrum distribution characteristics, frontal parietal mismatch negative (MMN) wave characteristics, and the Inter trial phase locking consistency (ITPC) characteristics of the prefrontal cognitive cortex and parietal motor cortex. In this study, 18 subjects were hired to complete the motor imagery tasks, induced by auditory stimuli of task related verbs and task independent nouns.

**Results:**

EEG power spectrum analysis showed that the activity of the contralateral motor cortex was significantly increased under the stimulation of verbs, and the amplitude of mismatch negative wave was also significantly increased. ITPC is mainly concentrated in μ, α, and γ bands in the process of motor imagery task guided by the auditory stimulus of verbs, while it is mainly concentrated in the β band under the nouns stimulation. This difference may be due to the impact of auditory cognitive process on motor imagery.

**Discussion:**

We speculate that there may be a more complex mechanism for the effect of auditory stimulation on the inter test phase lock consistency. When the stimulus sound has the corresponding meaning to the motor action, the parietal motor cortex may be more affected by the cognitive prefrontal cortex, thus changing its normal response mode. This mode change is due to the joint action of motor imagination, cognitive and auditory stimuli. This study provides new insight into the neural mechanism of motor imagery task guided by auditory stimuli, and provides more information on the activity characteristics of the brain network in motor imagery task by cognitive auditory stimulation.

## 1. Introduction

Sensory processing is one of the principal functions of the human body. Our senses can provide us with a continuous flow of information, which allows us to obtain the constantly changing environmental information around us. The input of environmental information plays an important role when a human completes some sport tasks (Li et al., 2020). Daily motion tasks, vision, hearing, and touch are the most important input of information flow to complete motion control. Amongst them, there are noticeable differences between auditory information input and the other two kinds of information input. Vision and touch belong to the intuitive information flow, they can make us intuitively understand the position and strength of objects and other information, while auditory input is often accompanied by prior knowledge learning. After receiving some meaningful or preset auditory information input, the brain will process it at the cognitive level. For example, the rhythmic sound stimulation (Song et al., 2015; Massimiliano et al., 2016), which is widely used in the rehabilitation of the lower limbs, will let the patients move the lower limbs rhythmically according to the specific prompt sounds in the treatment process. In fact, these prompt sounds are given special significance. After patients hear the syllable prompt. The brain will carry out cognitive processing analysis on them, which is also the unique feature of auditory stimulation (Thaut et al., 2007). Auditory stimuli can be divided into the following types according to the content of the information package: (1) Pure sound without meaning, such as noise, background sound, etc.; (2) environmental sound, such as finger knocking, footsteps, etc.; (3) semantic sound with meaning.

At present, the research on auditory stimulation is mainly based on ERP. In the study of the auditory ERP, mismatch negativity (MMN) can be used to detect some discernable changes caused by auditory stimuli, which is a reliable test method (Näätänen et al., 2007, 2010). Previous studies have extended the concept of MMN to higher-level cognitive processes, such as those involving semantics, which may have different response patterns in sound stimulation (Tsolaki et al., 2017). Therefore, in order to explore the effect of sound stimulation on motor intention, we need to know the difference of response mode between pure tone stimulation and verbs stimulation in human brain. The researchers found that there are different response patterns for pure tone stimulus and verbs. At present, the common conclusion is that a pure tone is mainly recognized in the right hemisphere, while meaningful tone is recognized in the left hemisphere. Satoko Asano studied whether segmented speech is regarded as a time unit like pure tone, and whether there is a difference between left and right ear stimulation. Twenty-five right-handed men were used to detect the time integral of segmenting speech by using a series of mismatched negative abnormal sounds in standard speech without paying attention to it, and unilateral and bilateral speech stimulation were performed, respectively (Asano et al., 2015). They found that all bilateral stimuli induced clear MMN with similar peak latency. At the same time, the amplitude of MMN was markedly different between pure tone stimulus and speech stimulus. Michael Weigl found that the left frontal anode tDCS could reduce the duration and intensity deviation of MMN by stimulating the subjects’ left prefrontal dorsolateral cortex with tDCS, which further highlighted the contribution of frontal lobe brain area to MMN production (Weigl et al., 2016). In addition, MMN data show that MMN can show people’s activation and attention conversion after receiving auditory stimulus stream, and can establish brain response process. Previous studies have further explored the effect of sound stimulation on motor function. Hauk et al. (2006) explored the activation dynamics of human motion recognition system. They studied the electrophysiological differences between the brain response to the sound produced by human finger movement (Hauk et al., 2006). They found that finger induced clicks produced more MMN than control stimuli. This shows that the sound stimulation related to movement can activate the memory track of familiar sound related to movement. In addition, Hauk et al. (2006) found that the MMN map of this incubation period showed a difference in brain response to natural finger and natural tongue sounds. Sound source estimation revealed that the click caused by fingers originated from the left hemisphere. The results show that: (1) the production of MMN is related to the process of bilateral temporal response and frontal parietal response; (2) external intervention can change the response mode of MMN; (3) hearing the environmental sound related to movement will arouse the activity of the movement area related to the dominant hand. Generally speaking, most of the previous studies focused on the activation of MMN effect by sound stimulation, while few people studied the effect of sound stimulation on human motor function.

In the research of motor imagination and brain-computer interface, almost all of these methods used 8–30 Hz band-pass filters, which was a relatively broad frequency band (Chen et al., 2021; Pei et al., 2021, 2022). Previous ERP studies on auditory stimulation suggested that some special frequency band concussions were caused by the noise of EEG acquisition system or uncontrollable interference source. However, recent studies have found that in some special frequency bands, such as α, β, and γ bands, the ongoing EEG oscillations are also related to stimulation related brain activities, which cannot be simply considered as “noise” (Engel et al., 2001; Jansen et al., 2003). Therefore, the use of ERPs alone in cognitive research may ignore some valuable information, such as the phase of oscillation in EEG. In order to solve this problem, researchers have developed several tools (Debener et al., 2006; Blankertz et al., 2007) to analyze the ongoing EEG dynamics, one of which is the inter-trial phase-locking consistency (ITPC), also known as “phase locking factor.” ITPC is a synchronous time-frequency measurement method related to specific experimental stimuli. It can be used to explore the relationship between EEG evoked potential and spontaneous EEG oscillation phase (Busch et al., 2009), and predict some cognitive processes. Papenberg et al. (2016). Found that the consistency of ITPC increased gradually from childhood to early adulthood, and then decreased from early adulthood to old age by comparing the ITPC between young and old people at low theta frequency band. Yu et al. (2018). Found that the components of auditory evoked potential driven by ITPC may coexist according to the different stages of information processing, which shows that ITPC plays a very important role in typical auditory evoked potential experiments, and it plays the role of amplitude prediction factor and difference discrimination factor.

Although there have been many studies on the effect of auditory stimulation on human cognitive function (Huang et al., 2018), the mechanism of auditory stimulation as feedback of motor task is not clear (Dijk et al., 2013). At the same time, the previous research methods are limited to single time domain or frequency domain phase research, and the correlation between time domain information and frequency domain phase information still lacks of exploration (Paavilainen et al., 2010). Therefore, we hope to further study how sound stimulation interferes with the neural mechanism of human motor function response. Based on the behavioral experiments of auditory stimulation and the above MMN and ITPC studies, we hypothesized that the auditory stimulation including meaningful (movement command) can affect the feedback control loop of brain in the motion imaging task, and the input of sound motion command stimulation can change the activation mode of the senior cortex in the motion imaging task. In order to verify this hypothesis, we try to analyze the relationship between temporal primary auditory cortex and motor sensory cortex in the process of auditory input and determine whether there are dominant factors. First of all, we use the traditional power spectrum topographic map method to obtain the brain’s regions activated by auditory stimulation under the motion imagination task. After determining the activation area, we used MMN method to study the effects of meaningful and meaningless auditory stimuli on the response patterns of the primary auditory cortex and advanced motor cortex of the temporal lobe in the motor imagination task. Then, in order to study the difference mechanism, we use ITPC method to obtain the frequency domain phase information in the response process, and analyze the possible sources of the difference, in order to verify the neural mechanism explanation of the effect of verbs stimulation on brain activation mode. Based on our findings, we infer that appropriate auditory training instruction in rehabilitation training may change the activation mode of the brain area. Therefore, this study can provide a new treatment for the rehabilitation of patients with motor disorders after stroke and other brain injury diseases.

## 2. Materials and methods

### 2.1. Participants

Eighteen healthy right-handed volunteers (mean age, 22; range, 19–24; 6 males and 12 females), with normal auditory function participated. All subjects’ semantic understanding is normal. All the subjects had signed informed consent and approval of the local ethics committee.

### 2.2. Experimental paradigm

**FIGURE 1:**
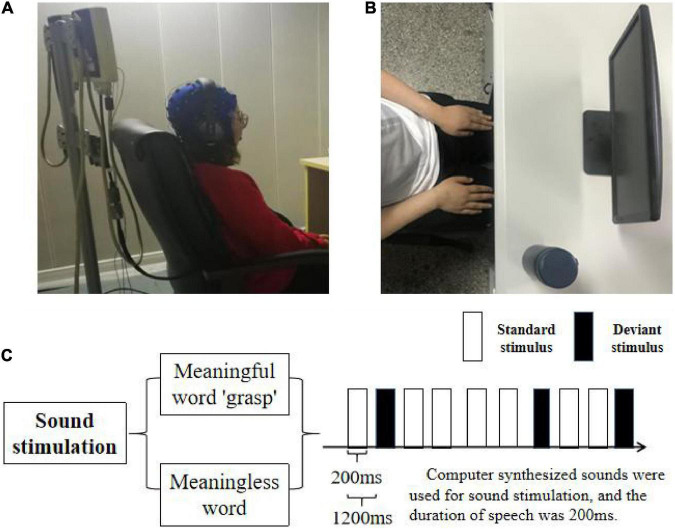
Task design. **(A)** The picture of the experimental environment, the subjects kept sitting relaxed with their hands flat on their legs. **(B)** The top view of the experimental environment, the subjects are facing the display device, and the physical cup for right hand movement imagination is placed on the desktop and on the right side of the display. **(C)** Experimental flow diagram, the experiment was divided into two groups. The two groups used the same standard stimulus, and the deviation stimulus used verbs “grasp” and no verbs, respectively. The stimulating sound was composed by computer. The playing time was 200 ms and the interval was 1,200 ms.

In this experiment, the subjects in the non-attention state were continuously presented with a standard stimulus and deviation stimulus, the probability of which was significantly different. Amongst them, the standard stimulus was pure tone stimulus, the tone length of which was the same as that of deviation stimulus, accounting for 90% of the total stimulus; the deviation stimulus was the meaningful “grasp” or meaningless word “grass” with similar pronunciation, accounting for 10% of the total stimulus, which was pseudo-random as shown in Figure 1C. The first five stimuli did not include biased stimuli, and at least five standard stimuli were designed between each two biased stimuli. At the beginning of the experiment, the subjects were asked to sit at the table in a comfortable way with their arms flat in front of the table. During the experiment, a cup will be placed in front of the right hand of the subject, and the subject is required to complete the movement imagination task of the right hand grasping when hearing the deviation stimulus “grasp.” During the whole experiment, the subjects have an obligation to look at the screen and keep the cup in the peripheral(side) vision. In this approach, in order to eliminate the influence of attention factors on the experimental results, the subjects can complete the grasping motion imagination task. The experiment consisted of two tasks: “M-ST” and “N-ST.” Amongst them, “M-ST” meant the deviation stimulus is the word with meaning of “grasp,” and “N-ST” meant the deviation stimulus is the word without meaning.

After the start of the experiment, each group contained 400 stimuli, which were played automatically according to the oddball paradigm. The interval between the stimuli was 800 ms, and each group was rested for 5 min after the end of the experiment. During the broadcast, the subjects wore monitoring headphones (iron triangle ath-m50x) and watched a silent movie. The subjects were required to maintain emotional stability, focus on the film, ignore the sound stimulation, and ask the subjects to the summary of film clips after the experiment to confirm their concentration. The overall program is shown in Figure 1A and the actual scene is shown in Figure 1B. The design and implementation are implemented by MATLAB software.

### 2.3. Recordings

The experiment was performed in an electromagnetic shielding room. EEG data was recorded with a SynAmps^2^ system (Neuroscan, EI Paso, TX, USA). An elastic cap with 32 mounted Ag/AgCI electrodes were positioned according to the extended 10–20 system which was used to detect EEG activities with the reference at the vertex. The EEG electrode impedances were kept below 5 kΩ during the entire experiment. All data were referenced against an electrode centered on the midline between Fz and Cz. EEG data were band-pass filtered (0.5–100 Hz). All signals were sampled to disk at 1,000 Hz together with event markers and saved on a hard disk for off-line analysis.

### 2.4. Data analysis

During the experiment, playing audio through MATLAB completes the alignment with EEG signal at the same time. At the same time considering the importance of time window selection in the motor imagery task (Jiang et al., 2020), we did not eliminate the 200 ms of the sound stimulus playback process on the EEG data segmentation to avoid missing critical information. Firstly, EEG is preprocessed by using the EEG toolbox, which mainly completes the 0.5–50 Hz band-pass filtering, which contains all the band information needed to be observed in this experiment. Then independent component analysis (ICA) is performed to remove the components with dominant artifacts (such as blink, eye movement, muscle artifacts, heartbeat). After pre-processing by the EEG toolbox, the EEG data is transformed into the data structure applicable to the field trip toolbox through the built-in interface function between the EEG toolbox and the field trip for subsequent processing and calculation.

To capture the difference in motor sensory cortex during the action execution period and momentary stabilization stage in different brain rhythms, power, MMN, and ITPC were estimated pair-wise between all EEG channels. Our study mainly used an open-source toolbox, Field-Trip (Oostenveld et al., 2011), for neurophysiological data analyzing.

### 2.5. Power estimated of EEG

The power spectrum of the electroencephalogram (EEG) signal of the interested electrode was calculated by using the multi-taper method, and the power of EEG was normalized to the sum of the total power of all frequencies (from 0 to 500 Hz). This is an estimate of the power ratio for a given frequency band. In the 1,200 ms test data, the EEG data after sound stimulation (200 ms after the start of the experiment) were used for further analysis. After grabbing the concerned data segments, the obtained data are divided into α/μ rhythms (5–14 Hz), β rhythms (14–35 Hz) and low γ rhythms (35–45 Hz) according to brain rhythms. When EMG signal or other artificial noise pollutes EEG signal, all EEG data segments are excluded artificially.

### 2.6. Estimation of MMN

The calculation of MMN is actually the difference wave obtained by subtracting the ERP signal generated by standard stimulus from the ERP signal generated by deviating stimulus. In general, the peak value of MMN occurs between 150 and 200 ms. In this paper, the starting time of acoustic stimulation is taken as the data starting point, and the data 200 ms after the start of acoustic stimulation is taken as the analysis time process. After data collection, the biased stimulus sequence and the standard stimulus sequence were overlapped and averaged, respectively. Finally, the MMN value is obtained by calculating the difference of the mean value.

### 2.7. Estimation of ITPC

Trial-by-trial time-frequency analysis was performed in EEGLAB (Makeig et al., 2004). Inter-trial phase coherence (ITPC) was computed using the “newtimef” function:


$$\text{ITPC}\,\left(f,t\right)=\frac{1}{n}\,\sum_{k=1}^{n}\frac{F_{k}\,\left(f,t\right)}{\left|F_{k}\,\left(f,t\right)\right|}.$$

In this function, *F*_*k*_(*f*,*t*) is the spectral estimate of trial k at frequency *f* and time *t* obtained using short-time Fourier transformation (STFT), and | | represents the complex norm of trial k. The modified STFT (with Hanning tapers) in EEGLAB uses overlapping sliding windows that are adaptive to the target frequency bins (i.e., the time window decreases linearly as frequency increases), which is recommended to overcome limitations of conventional fixed window in estimating low frequency activities. The frequency range analyzed was 0.5–50 Hz. Zero-padding was applied to windows without a sufficient number of sample points with a pad-ratio of 16 with a frequency spacing of 0.5 Hz. ITPC value of a given frequency at a given time point can range from 0 to 1. Larger ITPC values indicate higher phase consistency across trials, and smaller values indicate lower consistency or larger neural ‘jittering’. For the calculation of theta ITPC, the ITPC data were first averaged across the frequencies for further processing (Yu et al., 2018).

For this study, we not only pay attention to the influence of syllable stimulation in time domain, but also to the characteristics of the induced phase locking, which represents the time locking and induced components in the verbs stimulus. Therefore, we calculated inter-trial phase coherence (ITPC) and converted it into Rayleigh Z score to explain the difference between the M-ST group and the N-ST group.

### 2.8. Statistical analysis

In order to evaluate our hypothesis, the statistical analysis process is divided into three steps. First of all, the differences of EEG power spectrum between the stimulus task of M-ST group and that of N-ST group were compared by one-way ANOVA. In order to compare the MMN changes of M-ST group and N-ST group, we used one-way AVOVA and LSD correction to test the MMN differences of the two experimental groups and the differences of different wave bands in each experimental group. The third step of statistical analysis is to use one-way ANOVA in different frequency bands to verify the difference between the M-ST and N-ST groups.

## 3. Results

### 3.1. Topographic map of EEG power spectrum

**FIGURE 2:**
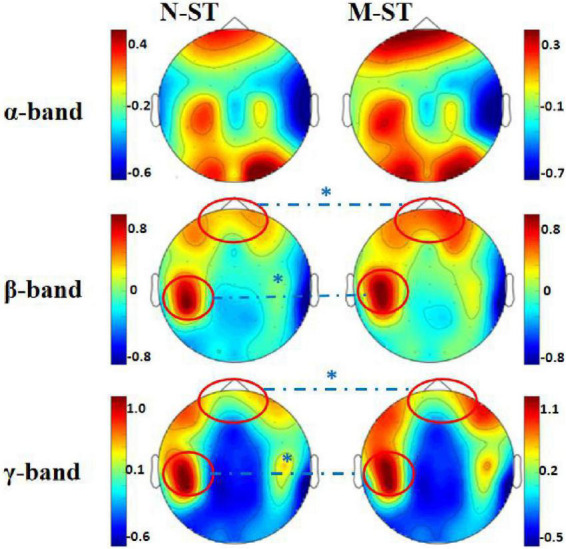
Mean EEG power spectrum of subjects. In the figure, the “N-ST” group is on the left and the “M-ST” group on the right. The EEG power distribution characteristics of α, β, and γ bands are shown, respectively. The red circle and “*” indicate the areas with significant differences in power spectrum statistics. It can be seen from the figure that the power produced by the prefrontal cortex and left motor cortex of the “M-ST” group was significantly higher than that of the “N-ST” group.

**FIGURE 3:**
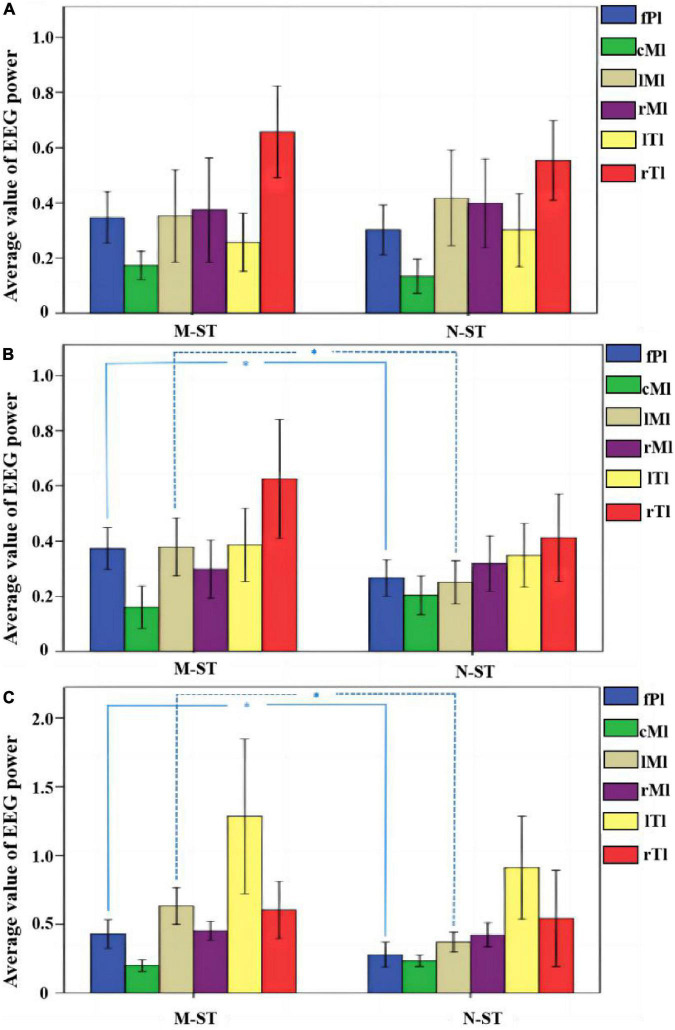
Average normalized EEG power across all the 18 subjects for M-ST task and N-ST task. **(A)** The mean normalized EEG power within the alpha band. **(B)** The mean normalized EEG power within the beta band. **(C)** The mean normalized EEG power within the gamma band. *Refers to *p* < 0.05 with one-way ANOVA. The statistical results showed that the activity of FPL and LML area was significantly increased in bate band and gamma band.

By comparing the distribution characteristics of EEG power and power spectrum map under the stimulation of nouns and verbs, we can determine whether the verbs stimulation has a differential effect on the brain activity under the exercise imagination task. We divided the data into α band, β band, and γ band to analyze the activity characteristics of primary auditory cortex and advanced motor cortex in different frequency bands. As shown in Figures 2, 3, we found that the main areas of brain activation in the motor imagination task under auditory stimulation are left temporal lobe and frontal parietal lobe. Under the verbs stimulus, the activation range of the left frontal parietal lobe was significantly stronger than that of the nouns stimulus, within the band of beta and gamma (*F* = 4.512 *p* = 0.041, *F* = 5.508 *p* = 0.031) (Table 1). At the same time, the activation level of cognitive cortex in the verbs stimulation group was higher than that in nouns stimulation group (*F* = 4.561 *p* = 0.040, *F* = 5.002 *p* = 0.032) (Table 1).

**TABLE 1:** Statistical analysis of EEG power in different brain regions and frequency bands.

Area	Band								
	**Alpha**			**Beta**			**Gamma**		
	***F***	**df**	***P*-value**	***F***	**df**	***P*-value**	***F***	**df**	***P*-value**
fp1	0.547	1	0.464	4.561	1	0.040	5.002	1	0.032
cM1	0.106	1	0.747	0.061	1	0.807	1.459	1	0.235
lM1	0.722	1	0.401	4.512	1	0.041	5.508	1	0.031
rM1	0.236	1	0.630	0.060	1	0.808	2.468	1	0.125
lT1	0.711	1	0.405	0.041	1	0.840	1.232	1	0.275
rT1	0.154	1	0.697	0.003	1	0.957	0.280	1	0.600

Statistical analysis in EEG power spectrum obtained by one-way ANOVA. fpl, prefrontal lobe; cMl, parietal motor cortex; lM1, left motor parietal lobe; rM1, right motor parietal lobe; lT1, left auditory temporal lobe; rT1, right auditory temporal lobe. Red in the table represents areas with significant differences.

### 3.2. Mismatch negativity (MMN)

**FIGURE 4:**
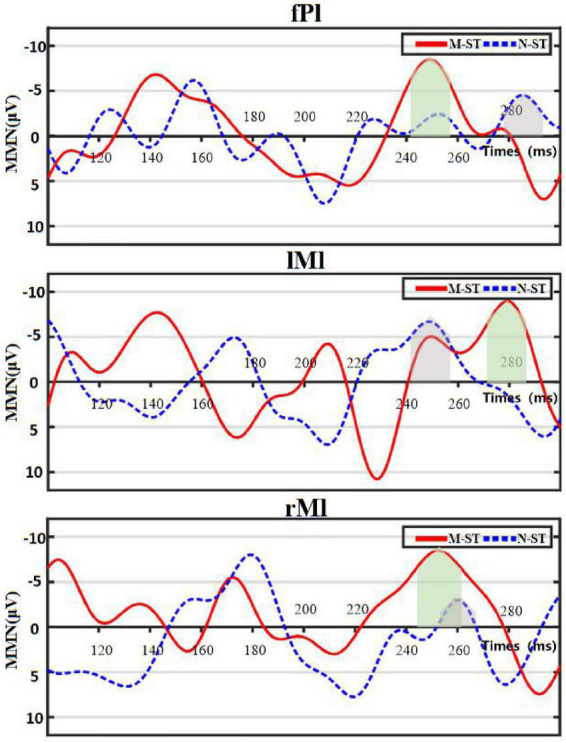
Figure of mean distribution of MMN. “fPl” represents the prefrontal parietal lobe area, “lMl” represents the left motor cortex, and “rMl” represents the right motor cortex. The red solid line represents the MMN value of group “M-ST”, and the blue dotted line represents the MMN value of group “N-ST”. The light green area and the light gray area represent the superposition range of MMN statistical values of group “M-ST” and group “N-ST”, respectively.

We use the traditional power spectrum mapping method to determine that the main brain areas activated by auditory stimulation in motor imagery task are frontal parietal lobe, bilateral temporal lobe and prefrontal lobe. MMN data induced by biased stimulus were recorded and calculated on FCZ of frontal midline electrode in the M-ST and N-ST groups. On this basis, the following two factor variances are carried out for amplitude and latency, respectively. As shown in Figure 4, the time-domain distribution of the mean MMN of eighteen subjects can be seen from the figure that two MMN peaks are generated within 100–300 ms, which are located near 150 and 250 ms, respectively. Because of the difference in the time of peak occurrence, we use the maximum value of MMN produced by verbs stimulation or nouns stimulation in 100—300 ms as the center, and take the average value of the sum of the values within 10 ms from the center as the statistical calculation value.

**FIGURE 5:**
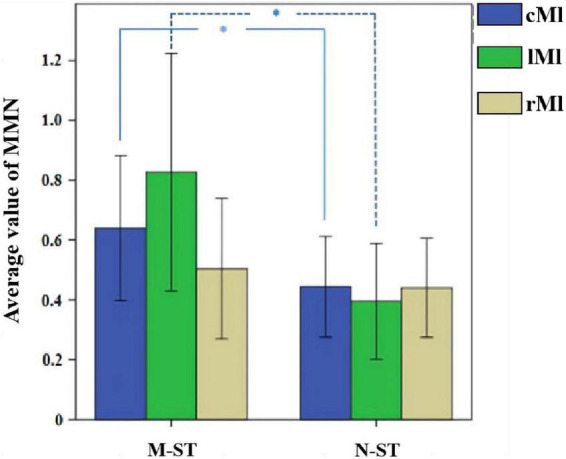
Mean MMN value across all 18 subjects and the results of statistical analysis. *Refers to *p* < 0.05 with one-way ANOVA. In the M-ST task, the MMN from the frontal parietal motor sensory cortex and the left motor cortex was significantly enhanced.

In this paper, one-way ANOVA was used to evaluate the effect of M-ST group and N-ST group on MMN amplitude. As shown in Table 2 and Figure 5, the MMN amplitude caused by “verbs stimulus” is significantly higher than that caused by “meaningless sound stimulus” (*F* = 4.495 *p* = 0.04).

**TABLE 2:** The statistical analysis result of MMN.

Area	MMN		
	***F***	**df**	***P*-value**
cMl	6.27	1	0.017
lM1	9.683	1	0.004
rM1	2.057	1	0.161

Statistical analysis in MMN value obtained by one-way ANOVA. cMl, parietal motor cortex; lM1, left motor parietal lobe; rM1, right motor parietal lobe. Red in the table represents areas with significant differences.

### 3.3. Inter-trial phase coherence (ITPC)

**FIGURE 6:**
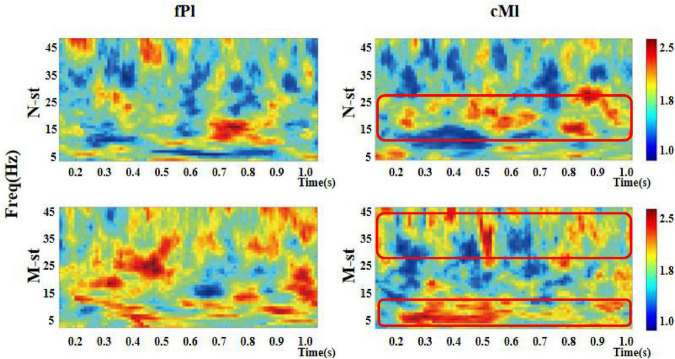
Time-frequency distribution of ITPC in prefrontal and parietal regions. “fPl” represents the prefrontal parietal lobe area, “cMl” represents the parietal motor cortex. “M-ST” represents the true meaning sound stimulation group, and the “N-ST” band represents nouns stimulation group. It can be seen from the figure that the ITPC values of the “M-ST” group in the frontal parietal lobe region are in a high state in all frequency bands. In the parietal motor cortex, ITPC values of M-ST group and N-ST group were different. The red boxes represent areas with high ITPC values.

**FIGURE 7:**
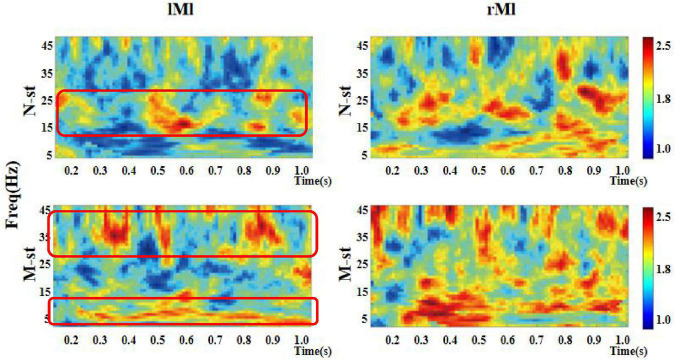
Time-frequency distribution of ITPC in left motor cortex and right motor cortex. “lMl” represents the left motor cortex and “rMl” represents the right motor cortex. “M-ST” represents the true meaning sound stimulation group, and the “N-ST” band represents nouns stimulation group. The distribution of ITPC values in left motor cortex was significantly different. There was no significant difference in the distribution of ITPC in right motor cortex.

**FIGURE 8:**
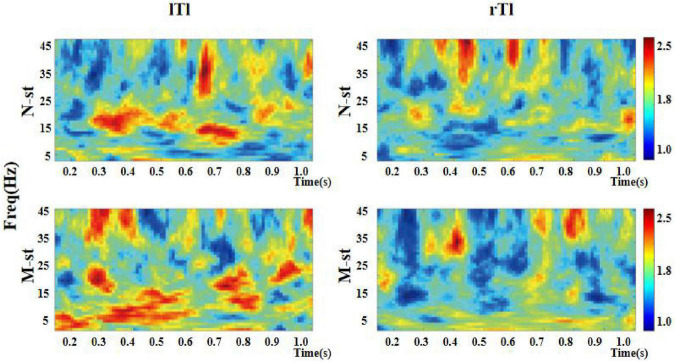
Time-frequency distribution of ITPC in left and right auditory temporal lobes. “lTl” represents the left temporal lobe and “rTl” represents the right temporal lobe. “M-ST” represents the true meaning sound stimulation group, and the “N-ST” band represents nouns stimulation group. As can be seen in the figure, the ITPC value of the left temporal lobe is higher than that of the right temporal lobe.

**FIGURE 9:**
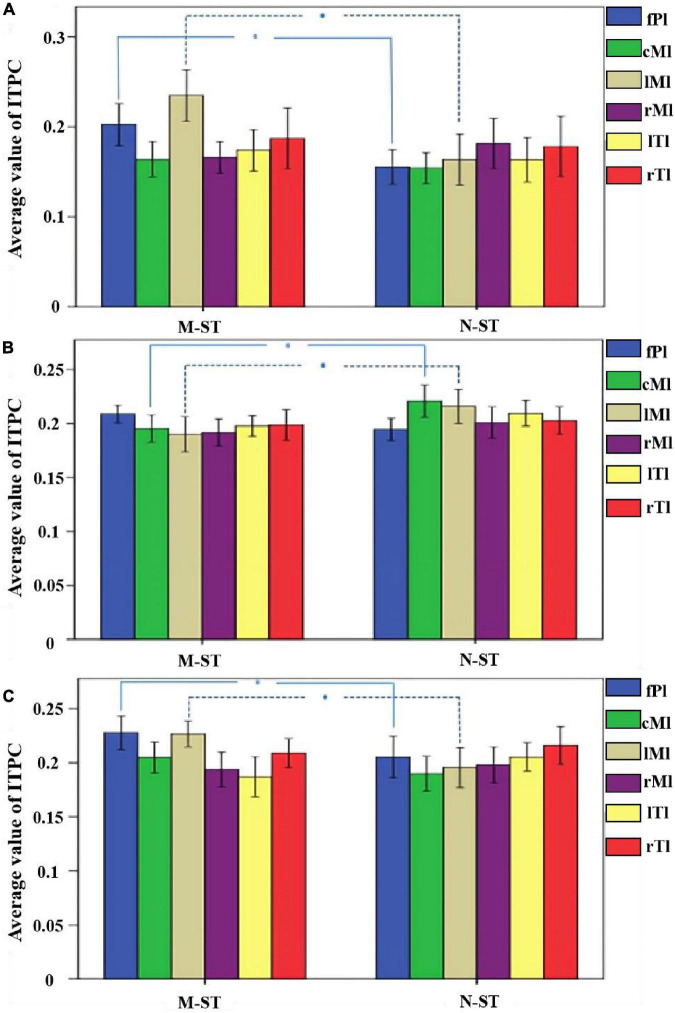
The mean ITPC values of 18 subjects in different brain regions and different frequency bands. **(A)** The mean value of ITPC within the alpha band. **(B)** The mean value of ITPC within the beta band. **(C)** The mean value of ITPC within the gamma band. *Refers to *p* < 0.05 with one-way ANOVA. The results of statistical analysis showed that the ITPC values of prefrontal cognitive cortex and left parietal motor cortex in M-ST group were significantly higher than those in N-ST group in alpha frequency band, the ITPC values of parietal motor cortex and left parietal motor cortex of N-ST group were significantly higher than those of M-ST group under bate frequency band, and ITPC values of prefrontal cognitive cortex and left parietal motor cortex of M-ST group under gamma band were significantly higher than those of N-ST group.

As a time-frequency measurement method, ITPC is mainly used to explore the relationship between EEG evoked potential and the phase of spontaneous EEG oscillation, and to show the auditory related cognitive process. As shown in Figures 6–8), in the beta and low gamma frequency bands, the distribution of ITPC is basically the same under the standard stimulation of M-ST and N-ST group, while the distribution of ITPC under the deviation stimulation is significantly different, as shown in Table 3. According to the distribution of EEG power spectrum, we mainly focus on the characteristics of ITPC in prefrontal and parietal motor cortex. The experimental results showed that the intra group phase coupling of prefrontal cortex showed a high level under the stimulation of verbs, while the intra group phase coupling of parietal motor cortex showed a significant frequency band differential distribution, as shown in Figure 9. It is characterized by the fact that ITPC is mainly distributed in the low gamma band and μ band under the stimulation of verbs, while ITPC is mainly distributed in the beta band under the stimulation of nouns.

**TABLE 3:** The statistical analysis result of ITPC.

Area	Band								
	**Alpha**			**Beta**			**Gamma**		
	***F***	**df**	***P*-value**	***F***	**df**	***P*-value**	***F***	**df**	***P*-value**
fp1	7.876	1	0.008	2.946	1	0.095	6.238	1	0.018
cM1	0.631	1	0.433	4.330	1	0.045	1.864	1	0.181
lM1	11.12	1	0.002	5.901	1	0.021	8.744	1	0.006
rM1	0.139	1	0.711	1.511	1	0.227	0.095	1	0.760
lT1	0.621	1	0.436	2.735	1	0.107	1.917	1	0.600
rTl	0.061	1	0.807	0.000	1	0.996	0.400		0.531

Statistical analysis in ITPC time-frequency superposition obtained by one-way ANOVA. fpl, prefrontal lobe; cMl, parietal motor cortex; lM1, left motor parietal lobe; rM1, right motor parietal lobe; lT1, left auditory temporal lobe; rT1, right auditory temporal lobe. Red in the table represents areas with significant differences.

## 4. Discussion

Based on the study of EEG power spectrum in the process of motor imagery task, we found that the right hand motor imagery task induced by auditory stimulation can cause a greater power response in the left parietal lobe (C3). This phenomenon is in line with the expectation of the experiment, and also consistent with the previous research results on the motion imagination task (Tabrizi et al., 2013; Burianova et al., 2014). At the same time, we compared the power intensity of the left and right motor cortex, the contralateral auditory temporal cortex and the prefrontal cognitive cortex under the stimulation of nouns and verbs, and found that the activation intensity of the left motor cortex under the stimulation of verbs was significantly higher than that of nouns. Similar findings have been found in other similar studies focusing on cortical activation. For example, Boe et al. (2014) pointed out in a study of visual guided motor imagery tasks in 18 healthy subjects that repeated motor imagery and neural feedback can produce lateralized effects on brain activity, and can improve the motor imagery task hand under the guidance of visual cues activation degree of contralateral motor cortex. Glover and Dixon (2013) studied visual stimulus guidance, context stimulus (visual context) guidance, and digital stimulus guided motor imagery tasks. They found that after removing visual guidance, context variables play a greater role in motor imagery tasks and behavior performance. This shows that context cognition plays an important role in sports imagination (Glover and Dixon, 2013). Based on the previous research results, we conclude that the motor imagination task under the stimulation of the verbs can enhance the activation intensity of the contralateral motor brain area similar to the visual stimulation. Compared with the research of Glover and Dixon (2013), the contextual stimuli used in our study come from auditory stimuli. At the same time, in our experiment, we also found that the prefrontal cognitive cortex is always in a high level of activated water products. Therefore, we speculated that visual and auditory contexts can guide the subjects to complete the task of motor imagination by affecting the cognitive cortex.

Previous studies have extended the concept of MMN to a higher-level cognitive process, such as the cognitive process involving semantics. Semantics may have different response patterns in sound stimulation. Therefore, in our experiment, we also studied the MMN amplitude intensity to explore the effect of sound stimulation on motor intention. The experimental results show that the MMN responses of both the M-ST and the N-ST groups are significant around 250 ms, and the MMN amplitude of the verbs stimuli is significantly higher than that of the nouns stimuli. Hauk et al. (2006) studied MMN induced by environmental sound stimulation related to finger movement and found that hearing the sound related to finger movement can arouse the activity of the motion area related to dominating fingers. According to the research data of Hauk et al., the motor regions in the human brain are part of the early automatic recognition of action related sounds. Lepage et al. (2010) had come to a similar conclusion. They believe that there is a rapid, multimodal resonance mechanism to regulate the activity of motor cortex after receiving sound stimulation. MMN is caused by any discernible change in a repetitive aspect of sustained auditory stimulation (Lepage et al., 2010). Even without attention, MMN can also cause the change of attention transfer, so it shows a significant response to the body. It is important that MMN reflects the function of NMDA receptor (Rosburg and Kreitschmann-Andermahr, 2015). The improvement of MMN can provide an effective index for the treatment of brain injury (Näätänen et al., 2019). The experimental results show that the MMN peak value produced by the meaningful stimulus is more advanced in the time axis than that produced by the meaningless stimulus, and the average amplitude is also significantly higher than that produced by the meaningless stimulus. Therefore, we believe that under the repeated verbs stimulus, it is more likely to cause the shift of attention of the subjects, making the subjects focus more on the movement imagination task, at the same time, the verbs stimulation has the similar function with the environment sound stimulation used by Hauk et al. (2006). It can also arouse the brain activity related to the hand movement.

Stroke and other neurological diseases have adverse effects on cognitive and motor functions. These damages are related to the changes of EEG power spectrum and event-related potential measurements. However, the effect of auditory stimulation on phase congruence (ITPC), a method to measure phase congruence during the experiment, is still unknown. ITPC is considered to reflect the ability of nerve response and related events to synchronize in time, so as to optimize information processing. In this study, we found that the phase consistency of auditory guided motor imagery evoked potential in different frequency bands of different brain regions showed a certain regularity in the motor imagery task under the stimulation of verbs compared with that under the stimulation of nouns. Our results showed that the ITPC in theta, α and low gamma rhythms increased significantly when the subjects were asked to perform motor imagery task under the effect of verbs stimulation. The ITPC frequency band differences in motion regions indicate that the task of motion imagination guided by sound stimulation is a complex “stimulus cognitive motion” feedback process, in which semantic recognition plays an important role. The increase of ITPC in the parietal motor cortex may be caused by attention or sensory stimulation, which was also found in previous studies on the effect of tactile stimulation on motor tasks. At the same time, the prefrontal cortex showed higher activation of ITPC under the stimulation of verbs, which indicated that the cognitive cortex could participate more in the activities of motor imagination tasks under the stimulation of verbs, and this stimulation improved the synchronization ability of prefrontal and sound stimulation events, which was completely consistent with the results of MMN. Papenberg et al. (2013) studied the difference of ITPC in theta/alpha frequency band in different age groups and found that ITPC in theta/alpha frequency band can represent the change of response time variability to stimulation, that is to say, the faster the response time, the higher the ITPC. Yu et al. (2018) also had similar research. They found that the ITPC amplitude of theta/alpha band was related to the decrease of auditory evoked response after the subjects were given verbal and non-verbal stimulation, respectively. In other words, verbal stimulation can improve the auditory evoked response (Yu et al., 2018). Combined with previous studies, we believe that the verbs stimulus can improve the response efficiency related to the exercise task in the exercise imagination task, that is to say, the verbs stimulus can reduce the response time of the subjects and improve the efficiency of the exercise imagination task. In the experiment, we found another interesting phenomenon: ITPC increased significantly in beta rhythm under the stimulation of no verbs, while ITPC showed a lower level in beta rhythm under the stimulation of verbs. This phenomenon has not been mentioned in similar studies. We speculate that this may be due to the lack of synchronization of cognitive process activated by nonsense stimulation, which results in the brain activity mainly concentrated in the parietal motor cortex during the task, and the main active rhythm in the traditional motor imagination task is beta rhythm.

The results showed that the ability of the brain to synchronize with the rhythmic stimulus was improved by the stimulation of the verbs. Therefore, the rise of ITPC may be an objective and mechanical measurement method of motor imagination function, which enables future work to study the relationship between brain world synchronization and specific brain function activation related to motor tasks.

## 5. Conclusion

This study has some limitations. First of all, we hope to understand the effect of auditory stimulation on motor function. Only from the results of motor imagery task, the verbs stimulation can effectively improve the brain response mode, but whether the verbs stimulation has similar results for the motor task remains to be verified. Therefore, in the future, we plan to carry out further research to confirm whether this improvement exists in motor tasks. Secondly, sample size is also a key limitation of our study. Small sample size may limit the statistical results and provide little classification information.

## Data availability statement

The raw data supporting the conclusions of this article will be made available by the authors, without undue reservation.

## Ethics statement

The studies involving human participants were reviewed and approved by Xi’an Jiaotong University Ethics Committee. The patients/participants provided their written informed consent to participate in this study.

## Author contributions

LL participated to study design, data collection and analysis, and manuscript writing. YZ participated to study design, data collection and analysis, and manuscript definition. LF participated to data analysis. JZ, CL, and JG participated in the data collection. JW and TL participated to study design. All authors read and approved the final manuscript.
